# Clinical efficacy of Atezolizumab/Bevacizumab combined with TACE in treating BCLC stage C hepatocellular carcinoma

**DOI:** 10.3389/fonc.2026.1744076

**Published:** 2026-03-19

**Authors:** Lei Yuan, Xiao-Hong Yang, Zhi-Li Ma, Ning Fan, Gen-Shu Wang, Yan-Ling Zhu

**Affiliations:** 1 Department of Hepatic Surgery and Liver Transplantation, Guangdong Provincial Hospital of Chinese Medicine, Guangzhou, Guangdong, China; 2 Department of Hepatic Surgery and Liver Transplantation, The Third Affiliated Hospital of Sun Yat-sen University, Guangzhou, Guangdong, China

**Keywords:** atezolizumab, BCLC stage C, bevacizumab, hepatocellular carcinoma (HCC), neutrophil-to-lymphocyte ratio (NLR)

## Abstract

**Background:**

The prognosis of advanced primary liver cancer [Barcelona Clinic Liver Cancer (BCLC) stage C] remains poor. Although atezolizumab plus bevacizumab (Ate/Bev) is a standard treatment, the potential of intensifying this regimen into a triple therapy by adding local intervention is unclear.

**Aim:**

To evaluate the efficacy of Ate/Bev plus transarterial chemoembolisation (TACE) in the treatment of BCLC stage C advanced hepatocellular carcinoma (HCC).

**Methods:**

The clinical data of 25 patients with BCLC-C HCC treated with Ate/Bev plus TACE at Guangdong Provincial Traditional Chinese Medicine Hospital between January 2022 and October 2024 were retrospectively analyzed. Efficacy was evaluated using Response Evaluation Criteria in Solid Tumors version 1.1, with follow-up until April 2025. The primary endpoints included the objective response rate, disease control rate, progression-free survival (PFS), and safety.

**Results:**

Of the 33 patients with intermediate-/advanced-stage HCC receiving the triple therapy, 25 were categorized as BCLC stage C HCC. Twelve patients died, and thirteen survived, during follow-up. The objective response rate was 32%, PFS was 7.960 ± 6.624 months, and overall survival (OS) was 12.880 ± 7.769 months. OS and PFS significantly differed by the neutrophil-to-lymphocyte ratio (≥ 3 vs < 3). The hazard ratios for median PFS and median OS were significantly different (P < 0.005) between the two groups, at 2.004 (95% confidence interval: 1.86–3.74) and 1.765 (95% confidence interval: 1.24–3.06), respectively. The incidence of all-grade adverse reactions was 100%, with grade 3/4 events seen in 4% of patients. No treatment-related deaths occurred.

**Conclusion:**

Atezolizumab combined with bevacizumab and TACE may be safe and effective in the treatment of BCLC stage C hepatocellular carcinoma. Baseline NLR may be an important potential biomarker for predicting the efficacy and prognosis of such patients. However, the above conclusions still need to be further verified by large-sample prospective randomized controlled trials.

## Introduction

Primary liver cancer is the one of the most prevalent malignancy and the third-leading cause of cancer death, with hepatocellular carcinoma (HCC) being the most common pathological type. In China, 70%–80% of HCC cases are diagnosed at intermediate or advanced stages, many of which are in Barcelona Clinic Liver Cancer (BCLC) stage C (1).

Recent studies have shown promising efficacy of triple therapy in specific HCC populations. For instance, the LEAP-012 study demonstrated that TACE combined with lenvatinib and pembrolizumab significantly improved progression-free survival compared with TACE alone in patients with unresectable, non-metastatic HCC, with a favorable trend in overall survival (2). However, whether this triplet strategy can be extended to more advanced stages—such as BCLC stage C HCC—and whether combining Ate/Bev with TACE yields similar benefits, remains uncertain.

The neutrophil-to-lymphocyte ratio (NLR) is the most extensively studied marker of systemic inflammation and is indicative of the host’s immune status. As an inexpensive and easily measurable parameter, NLR serves as a direct marker for systemic immune imbalance linked to bone marrow immunosuppression. With immunotherapy becoming a standard of care, NLR shows promise as a predictive biomarker. It may help identify likely responders to immunotherapy in hepatocellular carcinoma (HCC), thereby sparing non-responders from the burden of ineffective treatment (3).

This study retrospectively examined the efficacy and safety of triple therapy (Ate/Bev in combination with locoregional interventional therapy) in patients with BCLC stage C HCC accompanied by cirrhosis. It also probed into the influence of NLR on patient prognosis. The objective of this study is to offer evidence for formulating novel treatment strategies for specific subgroups of patients with BCLC stage C hepatocellular carcinoma, thus enhancing their prognosis.

## Materials and methods

### Subjects

The subjects were advanced HCC patients without surgical indications who received a triple therapy (Ate/Bev plus local intervention) at Guangdong Provincial Traditional Chinese Medicine Hospital from January 1, 2022, to April 30, 2024. Clinical treatment and follow-up data were collected.

The inclusion criteria were: (1) males and females aged ≥ 18 years; (2) clinically and/or radiologically diagnosed with HCC; (3) BCLC stage C HCC; (4) Child–Pugh class A or B; (5) Eastern Cooperative Oncology Group performance status of 0–2; and (6) expected survival of ≥ 3 months, along with good compliance and regular follow-up.

The exclusion criteria were: (1) prior treatments with immune checkpoint inhibitors (except Ate) or molecularly-targeted drugs (except Bev); (2) severe complications in other organs; (3) severe esophageal or gastric varices; and/or (4) allergy or intolerance to targeted or immunotherapy drugs.

### Monitoring and follow-up

Pretreatment monitoring: Before treatment, the patients underwent contrast-enhanced CT or magnetic resonance imaging scans, during which the baseline number and sizes of tumors were documented. A comprehensive systemic and liver function assessment, including blood tests, liver/kidney function tests, coagulation function testing, tumor markers, and immune parameters, was conducted.

Post-treatment monitoring: After completing the triple therapy, radiological assessment of tumors was performed every 6 wk (± 7 days). Tumor size and number were assessed and recorded according to Response Evaluation Criteria in Solid Tumors version 1.1. Laboratory tests (for blood, liver/renal function, coagulation, tumor markers, immune parameters, thyroid function, myocardial enzymes, and troponin) were performed every 3 wk.

Follow-up: Follow-up was carried out via personal communication and by reviewing the medical records up to April 30, 2025. Data including treatment-emergent adverse events, adverse events, time to disease progression or death, other anti-tumor treatments, and OS were obtained.

### Treatment

Digital subtraction angiography was performed via the femoral artery to locate HCC lesions and assess blood supply. A microcatheter was selectively placed into the tumor-feeding artery, avoiding the cystic artery branch. Post-lobaplatin (40 mg) infusion via the microcatheter, the tumor feeding artery was embolized with lipiodol suspension or drug-eluting microspheres. Post-embolization hepatic arteriography showed no tumor staining and feeding artery occlusion. Upon completion of the procedure, the puncture site was compressed for 15 minutes and pressure dressing applied. The affected limb was maintained in an upright position and immobilized for 8 hours, followed by 24 hours of bed rest. The puncture site was kept clean and dry. The treatment was performed once a month, and the procedure was repeated for 1–3 sessions based on the quantification of lipiodol deposition on follow-up CT scans.

Recommended doses of Ate and Bev were delivered every 3 wk until disease progression or intolerance.

### Endpoints and response evaluation criteria

The endpoints were categorized as primary and secondary. The primary endpoints included: (1) objective response rate (ORR): percentages of patients who achieve a complete response (CR) and a partial response (PR); (2) disease control [percentages of patients achieving a CR, PR, or stable disease (SD)]; (3) progression-free survival (PFS; time from initiation of treatment to disease progression or death from any cause); and (4) incidence of adverse drug reactions, graded by Common Terminology Criteria for Adverse Events version 5.0.

Efficacy was assessed according to Response Evaluation Criteria in Solid Tumors version 1.1 as follows: (1) CR, disappearance of all target lesions; (2) PR, ≥ 30% reduction of the sum of the longest diameters of target lesions compared to baseline; (3) progressive disease (PD), ≥ 20% increase in the smallest sum recorded or appearance of new lesions; and (4) SD, neither sufficient shrinkage to qualify for PR nor a sufficient increase to qualify for PD.

### Statistical analysis

Statistical analysis was performed using SPSS 25.0 (IBM Corp., Armonk, NY, USA). Both the *t*-test and χ^2^ test were applied. Survival curves were plotted using the Kaplan–Meier method, and OS was compared between groups with the log-rank test. Only univariate analysis was conducted due to the small sample size of BCLC stage C patients (n=25), which limited the statistical power of multivariate analysis. A P value (two-sided) of < 0.05 was regarded statistically significant.

## Results

### Patients and treatments

In total, 25 patients with BCLC stage C, inoperable HCC received a triple therapy of Ate/Bev plus local intervention. The mean age was 51.2 ± 12.47 years. All the baseline clinical data are shown in **Table 1**. All the patients had histories of HBV-induced cirrhosis, portal vein tumor thrombosis, and ≥ 3 intrahepatic lesions.

**Table 1:** Baseline patient characteristics and univariate analysis.

Characteristics	*N* = 25	*P* value
Gender		
Male	22	0.9501
Female	3	
Age, yr	51.2 ± 12.47	–
ECOG PS		
0	20	0.7938
1	5	
Child–Pugh		
A	22	0.3392
B	3	
Tumor size, cm		
< 10	9	0.2878
≥ 10	16	
Tumor number		
> 3	25	–
PVTT		
vp3	12	0.9189
vp4	13	
AFP ng/mL		
< 400	10	0.8408
≥ 400	15	
NLR		
< 3	8	0.0225*
≥ 3	17	

PVTT, Portal Vein Tumor Thrombus, AFP, Alpha-Fetoprotein, NLR, Neutrophil-to-Lymphocyte Ratio. "*" indicates a statistically significant difference with P < 0.05.

### Efficiency

The therapeutic responses were generally good among the 25 BCLC stage C HCC patients treated with Ate/Bev plus TACE: CR, *n* = 1 (4%); PR, *n* = 7 (28%); SD, *n* = 10 (40%); and PD, *n* = 7 (28%). The ORR was 32%, and the disease control rate reached 72% (**Table 2**).

**Table 2:** Survival analysis.

Survival Measure	Mean ± SD, months	Media, months	95% CI (LL)	95% CI (UL)
PFS	7.960 ± 6.624	6.000	5.364	10.556
OS	12.880 ± 7.769	12.000	9.835	15.925

LL, Lower Limit of the confidence interval; UL, Upper Limit of the confidence interval.

Of the 25 advanced HCC patients on triple therapy, 2 achieved imaging-suggested PR and underwent liver transplantation CR was pathologically confirmed after the surgery in one of these cases, and PR was confirmed in the other case. The rejection-free washout periods lasted > 50 days in both patients, during which no rejection or treatment-related complications were observed (4).

The PFS was 7.960 ± 6.624 months, and the OS was 12.880 ± 7.769 months, among the BCLC stage C HCC patients on triple therapy. The large standard deviations of PFS and OS indicate high heterogeneity in treatment outcomes, which may be attributed to three main factors: (1) Individual differences in tumor characteristics (e.g., tumor size, number of intrahepatic lesions) and liver function status among patients; (2) Variable responses to immunotherapy and anti-angiogenic therapy in different patients; (3) The small sample size that magnifies the impact of individual extreme survival outcomes on the overall data variability.

**Figure 1:**
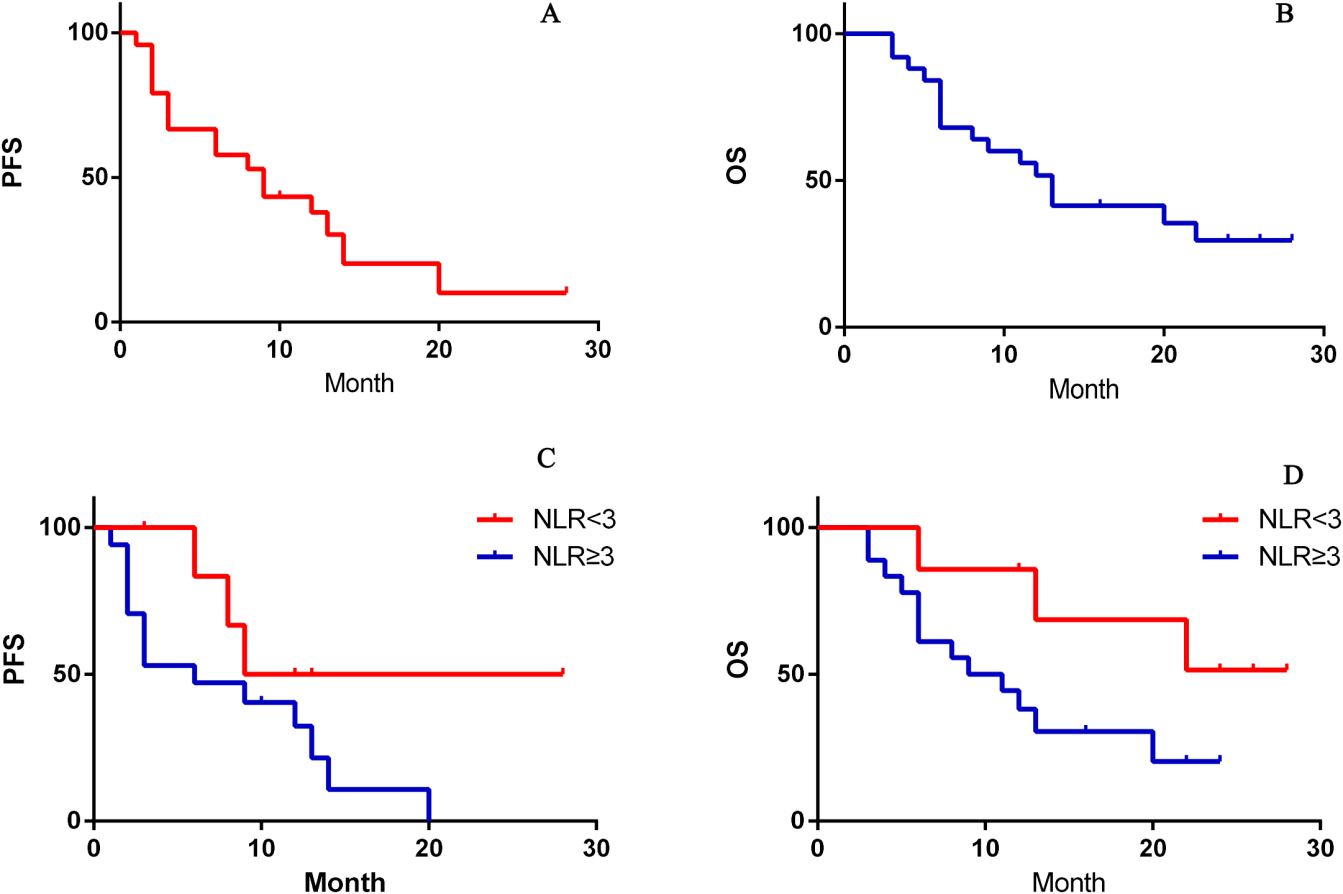
**(A, B)** Efficacy (OS and PFS) of atezolizumab/bevacizumab plus interventional therapy in BCLC stage C hepatocellular carcinoma, **(C, D)** stratified by the neutrophil-to-lymphocyte ratio (NLR) (≥ 3 *vs* < 3).

Univariate analysis identified NLR as the only statistically significant risk factor. OS and PFS differed significantly between the NLR ≥ 3 and NLR < 3 groups(**Table 1**, **Figure 1**). The hazard ratios for PFS and OS were 2.004 (95% confidence interval: 1.86–3.74) and 1.765 (95% confidence interval: 1.24–3.06), showing statistically significant differences. The two groups were generally similar in baseline clinical characteristics except for liver function. Despite three patients in the NLR < 3 group having Child–Pugh class B liver function, their prognoses were still better than those of the NLR ≥ 3 group patients (**Table 3**).

**Table 3:** Clinical data of 25 patients stratified by the neutrophil-to-lymphocyte ratio (NLR) (≥ 3 vs < 3).

Characteristics	NLR ≥ 3	NLR< 3	*P* value
Gender			
Males	14	8	0.205
Females	3	0	
Age, yr	50.471 ± 11.560	52.750 ± 14.945	0.679
ECOG PS			
0	14	6	0.668
1	3	2	
Child–Pugh			
A	17	5	0.007**
B	0	3	
Tumor size, cm			
< 10	4	6	0.304
≥ 10	13	2	
PVTT			
vp3	10	2	0.424
vp4	7	6	
AFP ng/mL			
< 400	7	3	0.861
≥ 400	10	5	

PVTT, Portal Vein Tumor Thrombus, AFP, Alpha-Fetoprotein, NLR, Neutrophil-to-Lymphocyte Ratio. "***" indicates a statistically significant difference with P < 0.01.

### Safety

Common adverse reactions included fever, abdominal distension, local treatment-related pain, chemotherapy-induced bone marrow suppression, abnormal liver function, thyroid dysfunction, and hypertension. Symptoms improved with the use of antipyretics, analgesics, leukocyte stimulators, liver-protective drugs, and/or antihypertensives, and with decreased doses of targeted drugs. The incidence of grade 3/4 adverse events was 4% (1/25), which was grade 3 hypertension in one patient; this adverse event was managed by a 25% dose reduction of Bev and the addition of a second-line antihypertensive agent, with complete resolution of hypertension within 1 week. For treatment discontinuations, only one patient discontinued Bev permanently due to grade 2 upper gastrointestinal bleeding after successful endoscopic hemostasis; no other patients discontinued Ate/Bev or TACE due to adverse events, and all patients completed the planned treatment courses as tolerated.

No treatment-related deaths occurred during the study period. All 25 patients experienced at least one adverse event. Bev was discontinued in one patient due to upper gastrointestinal bleeding after endoscopic hemostasis. Notably, the accuracy of the adverse reaction data might be limited by the retrospective design.

## Discussion

This study assessed the real-world efficacy and safety profile of atezolizumab-bevacizumab (Ate/Bev) in a distinct subgroup of Chinese patients with hepatocellular carcinoma. Among the 25 enrolled patients diagnosed with BCLC stage C HCC, the ORR was 32% and the median PFS was 6 months, which aligned with the findings of the IMbrave150 study (5). All of our patients had hepatitis B virus (HBV)-induced cirrhosis complicated by portal vein tumor thrombus, and their OS was somewhat poorer compared to that in the IMbrave150 study. the observed mortality rate suggests that the antitumor effect of triple therapy may be temporary in many patients, highlighting the need for optimizing patient selection and developing strategies to overcome acquired resistance. Although diagnosed with Barcelona Clinic Liver Cancer (BCLC) stage C hepatocellular carcinoma, these patients still achieved favorable progression-free survival and overall survival.

The first-line systemic therapy for BCLC stage C HCC is currently recommended as Ate/Bev combination therapy in the 2025 EASL Clinical Practice Guidelines on Hepatocellular Carcinoma, while the combination of systemic therapy with local interventions such as TACE is listed as a research direction without definitive clinical recommendations due to the lack of sufficient RCT evidence. Two ongoing phase III RCTs (IKF-035/ABC-HCC Study Design) are currently evaluating the efficacy and safety of Ate/Bev combined with TACE in advanced HCC patients, and our real-world study provides preliminary clinical evidence for these ongoing trials, which can lay a foundation for the design and interpretation of their results.

NLR is a biomarker of systemic inflammation measured from complete blood count. An elevated NLR can indicate neutropenia (a cancer-promoting chronic inflammation) but may also reflect lymphocytopenia resulting from impaired adaptive immune function mediated by lymphocytes. Regardless of treatment (surgery, transplantation, ablation, TACE, or systemic therapy), an elevated NLR is associated with poor prognosis in HCC (5–7). Retrospective studies suggest that, in the era of immunotherapy, NLR may predict efficacy and prognosis in advanced non-small cell lung cancer treated with first-line immune checkpoint inhibitors. Similarly, with dual immunotherapy (e.g., ipilimumab plus anti-PD-1/PD-L1) for HCC, NLR ≥ 3.1 was the only independent prognostic risk factor for OS (8). NLR changes might predict outcomes in advanced cancer patients treated with anti-PD-1/PD-L1 drugs (9, 10). The cut-off value of NLR = 3 identified in this study can help clinicians stratify the prognosis of patients receiving the triple therapy in advance: patients with NLR <3 have a significantly better PFS and OS, while those with NLR ≥3 are at high risk of poor treatment outcomes and require more frequent efficacy assessments (e.g., shortened radiological evaluation intervals from 6 weeks to 4 weeks). However, it should be emphasized that the predictive value of NLR in this study is based on a small single-center retrospective cohort, and further prospective multicenter studies with larger sample sizes are urgently needed to validate its cut-off value, predictive efficiency, and clinical utility in HCC patients receiving Ate/Bev+TACE triple therapy. In addition, the dynamic changes of NLR during treatment may also have predictive value, which will be the focus of our subsequent research.

This study has several notable limitations that should be fully considered when interpreting the results. First, the retrospective study design may introduce selection bias (e.g., the selection of patients with good compliance and expected survival ≥3 months), which compromises the causal inference between the triple therapy and clinical outcomes. Second, the small single-center sample size (n=33, 25 for BCLC stage C) reduces the statistical power of the study and limits the generalizability of the results to the broader HCC population; in addition, the small sample size only allowed for univariate analysis, and multivariate analysis to identify independent prognostic factors could not be performed. Third, the absence of an Ate/Bev monotherapy control group makes it impossible to directly attribute the observed clinical benefits solely to the intensification of TACE, and the results can only reflect the real-world efficacy of the triple therapy rather than a head-to-head comparison with the standard first-line regimen. Fourth, the short follow-up period (until April 2025) limits the assessment of long-term survival outcomes and late adverse events of the triple therapy. Fifth, the retrospective collection of adverse event data may lead to incomplete reporting of mild adverse events (e.g., grade 1 fatigue, nausea). Finally, all enrolled patients had HBV-induced cirrhosis and portal vein tumor thrombosis, which may lead to a relatively homogeneous study population and limit the applicability of the results to HCC patients with other etiologies (e.g., HCV, alcoholic liver disease).

## Conclusion

This study suggests that Ate/Bev in combination with TACE may offer clinical activity with a manageable safety profile in the early treatment of patients with BCLC stage C HCC in a real-world setting. Exploratory analyses further identified a potential correlation between baseline NLR and post-treatment survival. These findings raise the hypothesis that treatment response may warrant closer monitoring in HCC patients with elevated NLR to optimize management during Ate/Bev therapy. Given the hypothesis-generating nature of this study, these observations require confirmation through prospective investigations.

## Data Availability

The raw data supporting the conclusions of this article will be made available by Yuan Lei, leiy100798@163.com, without undue reservation.
